# Identification of a novel *AIFM1* variant from a Chinese family with auditory neuropathy

**DOI:** 10.3389/fgene.2022.1064823

**Published:** 2022-11-21

**Authors:** Rongrong Wang, Xiaohui Bai, Huiming Yang, Jingyu Ma, Shudong Yu, Zhiming Lu

**Affiliations:** ^1^ Department of Clinical Laboratory, Shandong Provincial Hospital, Shandong University, Jinan, China; ^2^ Department of Otolaryngology-Head and Neck Surgery, Shandong Provincial Hospital Affiliated to Shandong First Medical University, Jinan, China; ^3^ Department of Clinical Laboratory, Shandong Provincial Hospital Affiliated to Shandong First Medical University, Jinan, China

**Keywords:** auditory neuropathy, hearing loss, mutation, apoptosis inducing factor mitochondrion associated 1 (*AIFM1*), next-generation sequencing (NGS)

## Abstract

**Background:** Auditory neuropathy (AN) is a specific type of hearing loss characterized by impaired language comprehension. Apoptosis inducing factor mitochondrion associated 1 (*AIFM1*) is the most common gene associated with late-onset AN. In this study, we aimed to screen the pathogenic variant of *AIFM1* in a Chinese family with AN and to explore the molecular mechanism underlying the function of such variant in the development of AN.

**Methods:** One patient with AN and eight unaffected individuals from a Chinese family were enrolled in this study. A comprehensive clinical evaluation was performed on all participants. A targeted next-generation sequencing (NGS) analysis of a total of 406 known deafness genes was performed to screen the potential pathogenic variants in the proband. Sanger sequencing was used to confirm the variants identified in all participants. The pathogenicity of variant was predicted by bioinformatics analysis. Immunofluorescence and Western blot analyses were performed to evaluate the subcellular distribution and expression of the wild type (WT) and mutant *AIFM1* proteins. Cell apoptosis was evaluated based on the TUNEL analyses.

**Results:** Based on the clinical evaluations, the proband in this family was diagnosed with AN. The results of NGS and Sanger sequencing showed that a novel missense mutation of *AIFM1*, i.e., c.1367A > G (p. D456G), was identified in this family. Bioinformatics analysis indicated that this variant was pathogenic. Functional analysis showed that in comparison with the WT, the mutation c.1367A > G of *AIFM1* showed no effect on its subcellular localization and the ability to induce apoptosis, but changed its protein expression level.

**Conclusion:** A novel variant of *AIFM1* was identified for the first time, which was probably the genetic cause of AN in a Chinese family with AN.

## Introduction

Auditory neuropathy (AN) is a type of auditory disorder mainly characterized by impaired speech comprehension ability, which manifests as a group of specific syndromes in audiology, also known as the auditory neuropathy spectrum disorder (ANSD) (Starr et al., 1996; El-Badry and McFadden, 2009; Talaat et al., 2009).

The pathophysiology of AN indicates that the outer hair cells show normal function, whereas the inner hair cells and auditory nerves as well as their synapses are dysfunctional. The clinical manifestations include: (1) the degree of impaired speech recognition rate is inconsistent with the degree of hearing impairment, and the word comprehension ability and speech discrimination ability are poor, especially in noisy environment (Elrharchi et al., 2020); (2) the audiological examination is characterized by normal cochlear microphonic (CM) and meaningful otoacoustic emission (OAEs), but the auditory brainstem response (ABR) waveform is either missing or illegible in the patient (Starr et al., 1996). Studies showed that the prevalence rate of AN in neonates is 0.009%, and about 6.5% of the congenital sensorineural hearing loss is caused by AN (Boudewyns et al., 2016). AN is also one of the refractory diseases leading to hearing and speech communication disorders in both infants and adolescents, accounting for 10% of permanent hearing loss in children (Chinese Multi-center Research Collaborative Group on Clinical Diagnosis and Intervention of Auditory Neuropathy, 2022).

According to the age of onset, cases of AN are divided into two groups, i.e., congenital AN and late-onset AN. Studies have shown that the etiology of AN is complex, with about 48% as idiopathic, 10% related to various factors, such as ototoxic drugs and metabolic, immune, and infectious factors, and about 42% as genetic (Moser and Starr, 2016). There are three types of inheritance pattern for AN: autosomal recessive, autosomal dominant, and X-linked recessive (Wang et al., 2020). To date, among the over 20 genes associated with AN, apoptosis inducing factor mitochondrion associated 1 (*AIFM1*) is considered as the most prevalent gene associated with the late-onset AN and X-linked recessive inheritance mode (Zong et al., 2015; Wang et al., 2020).

First cloned and named in 1999, *AIFM1* is located in the Xq25-q26 region of human chromosomes (Susin et al., 1999). The *AIFM1* gene is 36.471 kb in length, containing a total of 16 exons and encoding a 613-amino acid apoptosis-inducing factor (AIF). The AIF is a flavin protein with oxidoreductase activity, composed of two flavin adenine dinucleotide (FAD) domains, a nicotinamide adenine dinucleotide (NADH) binding domain, a C-terminal domain with oxidoreductase activity, and a mitochondrial localization signal (MLS) at the N-terminal (Yuste et al., 2011). The *AIFM1* has two main functions. First, it is involved in the caspase-independent death effect when mitochondria are transferred to nuclei under apoptosis stimulation, resulting in caspase-independent programmed cell death (Joza et al., 2001). Second, as a NADH oxidoreductase that is dependent on FAD, *AIFM1* is crucial for oxidative phosphorylation, redox control, and respiratory chain activity in the organism (Ghezzi et al., 2010; Itai et al., 2011).

To date, a total of 19 *AIFM1* variants have been identified as pathogenic factors for AN (https://www.hgmd.cf.ac.uk/; accessed on 03 October 2022). It is conceivable that there are other *AIFM1* variants still remaining uncovered responsible for pathogenesis of AN. Therefore, in this study, we attempted to detect the pathogenic variants of *AIFM1* in a Chinese family with AN enrolled in our hospital and to further investigate the effects of this variant on AN development.

## Materials and methods

### Subjects

One patient and eight unaffected individuals from a Chinese family as well as a total of 200 Chinese individuals with no genealogical relationships (i.e., controls) were included in this work. A comprehensive clinical evaluation, including the disease history and the audiological tests, was performed on all participants. The audiological tests included the pure tone threshold (PTA), distortion product otoacoustic emission (DPOAE), auditory brainstem response (ABR), speech discrimination score (SDS), and auditory steady-state response (ASSR). The informed consent was signed by each participant prior to the clinical evaluation. The study was conducted in accordance with the Declaration of Helsinki with all procedures performed in accordance with the ethical standards of Shandong Provincial Hospital (Approval # SWYX: NO. 2021-511).

### Targeted next-generation sequencing

The genomic DNA was extracted from 1 ml whole blood using the Genomic Blood DNA Extraction Kit (Axygen, San Francisco, CA, United States) according to the manufacturer’s instruction. The proband’s DNA was diluted and then fragmented, ligated, amplified, and purified. GenCap^®^ deafness gene capture probe V4.0 and GenCap^®^ mitochondrial ring gene capture probe V1.0 (MyGenostics, Beijing, China) were used to capture the exon regions and the adjacent 20-bp intron regions of 406 genes of the proband. After elution, amplification, and purification, the captured regions were double-ended sequenced using Illumina HiSeq X TEN high-throughput sequencing platform (Illumina, United States). The detailed procedures of library preparation, sequencing, and bioinformatics analysis were described in our previous study (Xiao et al., 2016).

### Polymerase chain reaction (PCR) amplification and Sanger sequencing

PCR was performed in a 50-μl reaction mixture with the specific primers (forward primer 5′-TCC​CTT​TGT​ATG​AAG​CTA​ACT​GG-3′ and reverse primer 5′-CCA​TTA​CAA​GTG​TTC​TTT​GAG​CC-3′), as previously described (Bai et al., 2014). After the purification, the PCR products were sequenced by the ABI 3500 Genetic Analyzer (Thermo Scientific, Applied Biosystem, CA, United States). Nucleotide variations were identified by aligning the sequences with the *AIFM1* available (GenBank accession NM_004208.4) using the Chrome software.

### Pathogenicity assessment of *AIFM1* variant

The harmfulness of variant was evaluated with pathogenicity prediction tools and databases, including REVEL (Ioannidis et al., 2016), SIFT (http://sift.jcvi.org/), PolyPhen-2 (http://genetics.bwh.harvard.edu/pph2/), and Mutation Taster (http://www.mutationtaster.org/). The novelty of variant was determined by screening based on the 1000 Genomes (1000G) and Exome Aggregation Consortium (ExAC) databases as well as the literature and publicly available databases, i.e., the Deafness Variation Database (DVD) (http://deafnessvariationdatabase.org/; accessed on 03 October 2022) and the ClinVar database (http://www.ncbi.nlm.nih.gov/clinvar/; accessed on 03 October 2022).

A total of eight *AIFM1* protein sequences of eight animal species, including *Homo sapiens* (O95831), *Pan troglodytes* (K7BTY6), *Bos taurus* (A0A4W2I7F3), *Macaca mulatta* (F7C728), *Canis lupus familiaris* (A0A8C0JLH6), *Rattus norvegicus* (Q9JM53), *Mus musculus* (Q9Z0X1), and *Danio rerio* (Q5XFY2), were downloaded from the UniProt database to perform the multiple sequence alignment by the Clustal Omega online tools (https://www.ebi.ac.uk/Tools/msa/clustalo/; accessed on 03 October 2022) to assess the conservation of the *AIFM1* proteins.

### Modeling analysis of *AIFM1* protein structure

To determine the effects of *AIFM1* variant on the protein structure, the 3-dimensional (3D) molecular structures of human wild type (WT) and mutant *AIFM1* proteins were simulated using an automated homology modeling program I-TASSER (http://zhanglab.ccmb.med.umich.edu/; accessed on 03 September 2022) with the protein structure visualized by Swiss-PdbViewer 4.1.

### Cell culture

The Shandong Provincial Key Laboratory of Otology provided the HEK293 cells, which were cultured in the Dulbecco’s modified Eagle’s medium (DMEM) (Gibco, Grand Island, NY, United States) containing 10% fetal bovine serum (FBS) (Gibco, Grand Island, NY, United States) and 1% penicillin/streptomycin (Macgene, Beijing, China) with 5% CO_2_ at 37°C.

### Transient transfection and immunofluorescence analysis

The WT and mutant *AIFM1* plasmids expressing GFP-tagged (pCMV3-C-GFPSpark) were synthesized by BioSune Biotech (Jinan, China). Sequences of WT and mutant *AIFM1* plasmids were verified by Sanger sequencing. The HEK293 cells were planted onto the 24-well plates and grown to 60% confluence. The expression plasmids of WT and mutant *AIFM1* were transfected into cells by using Lipofectamine 3000 transfection reagent (Invitrogen, Waltham, MA, United States). In 48 h, the cells were fixed in 4% paraformaldehyde for 15 min, washed thrice with PBS, and then incubated with DAPI (D9542, Sigma-Aldrich, St. Louis, MO, United States) for 10 min in dark. Finally, the samples were washed three times with PBS. After sealing, the samples were observed and imaged through a Leica TCS SP8 confocal fluorescence microscope (Leica Microsystems, Biberach, Germany). The relative fluorescence intensity was quantified with ImageJ software.

### Western blot

Total protein was extracted from the HEK293 cells transfected with the WT and mutant *AIFM1* plasmids. The proteins were denatured and separated by 10% SDS-PAGE electrophoresis and then transferred to polyvinylidene fluoride (PVDF) membranes (ISEQ00010, Merck Millipore, China). After blocking, the membranes were incubated with mouse monoclonal anti-GFP antibody (Proteintech, Wuhan, China) and mouse monoclonal anti-β-actin antibody (ZSGB-Bio, Beijing, China), and followed by anti-mouse IgG conjugated horseradish peroxidase (ZSGB-Bio, Beijing, China). Finally, the immunoblots were detected with an Immobilon Western HRP Substrate kit (WBKLS0100, Millipore, Schaffhausen, Switzerland) using the enhanced chemiluminescence system (Tanon5200, Shanghai, China). The gray scale of each band was quantified with ImageJ software and normalized by β-actin.

### Tunel assay

The human HEK293 cells seeded in 24-well culture plates were transfected with plasmids of WT and mutant *AIFM1* by using Lipofectamine 3000 transfection reagent (Invitrogen, Waltham, MA, United States). In 48 h, the cells were fixed in 4% paraformaldehyde for 30 min and permeabilized with proteinase K (20 μg/ml) at room temperature for 5 min. Each sample was stained with 50 μl TUNEL TdT Enzyme working solution (Meilunbio, Dalian, China) for 60 min at 37°C. The fluorescence intensity of the sample was detected with EX at 546 nm and EM at 570 nm.

### Statistical analysis

Data presented as mean ± standard deviation (SD) of three biological replicates for each experiment were analyzed using one-way ANOVA (nonparametric or mixed) with Dunnett’s multiple comparison test and two-tailed unpaired t-test by Graphpad Prism (V8.3.0). The significant difference was set at the two tailed *p* value less than 0.05.

## Results

### Participants and clinical evaluations

**FIGURE 1:**
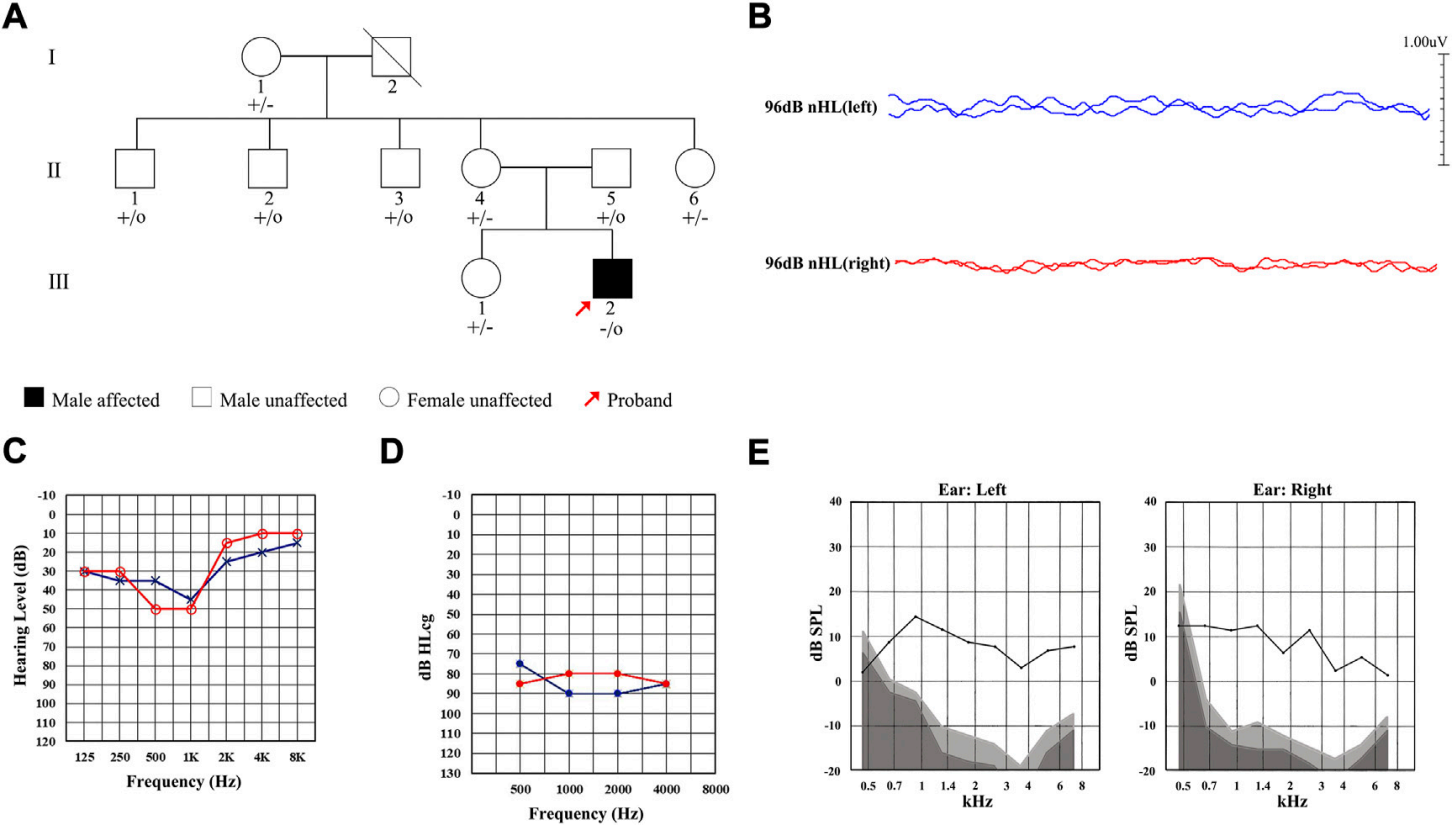
Pedigree and clinical characteristics of the Chinese family diagnosed with auditory neuropathy (AN). **(A)** Pedigree of the Chinese family with AN. Individuals harboring hemizygous (–/o) or heterozygous (+/–) *AIFM1* (c.1367A > G) mutation and WT (+/+) are indicated. The red arrow denotes the proband. The square indicates a male and the circle indicates a female. The hearing-impaired individual is indicated by a blackened square. **(B)** ABR thresholds of the proband. Red and blue lines represent the right and left ears, respectively. **(C)** PTA test of the proband. Red and blue lines represent the right and left ears, respectively. **(D)** ASSR thresholds of the proband. Red and blue lines represent the right and left ears, respectively. **(E)** DPOAE test results of the proband. Gray area indicates level of noise and the black line indicates the level of response.

A total of nine members of a Chinese family were included in this study, including one affected (Ⅲ-2) and eight unaffected (Figure 1A). The proband Ⅲ-2 was a 21-year-old male and complained of hearing loss in both ears for 6 years, capable of hearing sounds but not clearly, with occasional tinnitus, which was more pronounced in noisy environments. The results of ABR showed that there was no significant waveform change observed in both ears of the proband with the stimulated of 96 db (Figure 1B). The PTA test results showed that the proband had low-frequency mild hearing loss in both ears (Figure 1C) with the SDS in the left and right ears of 45% and 43%, respectively, showing a disproportionate decline in pure tone hearing. The ASSR test showed that its threshold was significantly higher than that of PTA (Figure 1D). Notably, the DPOAE response was completely preserved (Figure 1E). These results of audiological tests indicated that the proband was presented with typical symptoms of AN.

### Identification of a novel *AIFM1* variant

**FIGURE 2:**
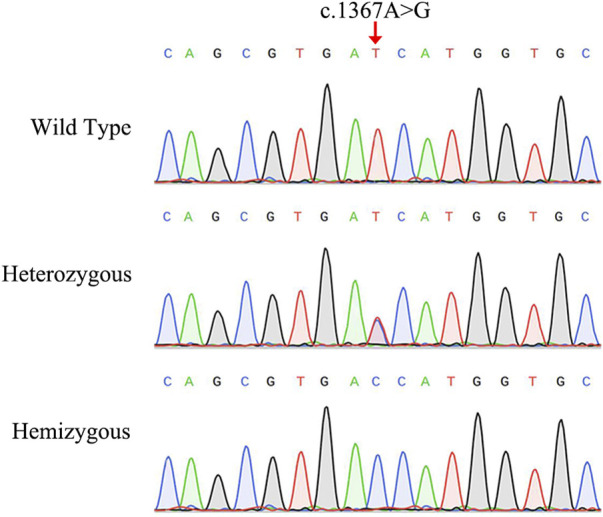
Sanger sequencing chromatograms of the Chinese family diagnosed with auditory neuropathy. The red arrow indicates the location of the *AIFM1* C.1367A > G variant.

The NGS analysis was performed based on the proband. The result showed that there was a base substitution (A to G) at position 1367 in the coding region of *AIFM1* gene. Then, the candidate variant was confirmed by Sanger sequencing (Figure 2), showing that the proband carried the c.1367A > G hemizygous variant, the proband’s sister and mother had the c.1367A > G heterozygous variant, and the proband’s father did not have the variant, indicating that the variant of c.1367A > G came from the mother, and the WT allele came from the father. We then performed Sanger sequencing on other members of the family and found that the variant was co-segregated with the phenotypes of AN in this family (Figure 1A) with the X-linked recessive inheritance pattern in this family. The results of Sanger sequencing also showed that this variant was absent in the 200 controls. Furthermore, this variant was not detected in neither ExAC nor 1000G databases and was not reported in the literature and publicly available databases, i.e., the DVD and the ClinVar databases (Table 1). These results indicated that the novel variant of *AIFM1*, i.e., the missense mutation c.1367A > G, was identified in the proband of this Chinese family in this study.

**TABLE1:** Characteristics of *AIFM1* variant, analysis of predicted protein structure and disease-causing effects based on various databases.

Gene	Variant	REVEL	SIFT	Polyphen-2	Mutation taster	ExAC	1000 G	DVD	ClinVar
*AIFM1*	c.1367A > G (p. D456G)	Probably damaging	Damaging	Damaging	Disease causing	Novel	Novel	Novel	Novel

Abbreviations: *AIFM1*, Apoptosis inducing factor mitochondrion associated 1; c, variation at cDNA level; p, variation at protein level; ExAC, Exome Aggregation Consortium; 1000 G, 1000 Genomes; DVD, Deafness Variation Database.

### Bioinformatics analysis of the pathogenicity of the *AIFM1* variant

The results of bioinformatics analysis showed that the variant c.1367A > G was predicted to be deleterious by REVEL, SIFT, Polyphen-2, and Mutation Taster (Table 1).

**FIGURE 3:**
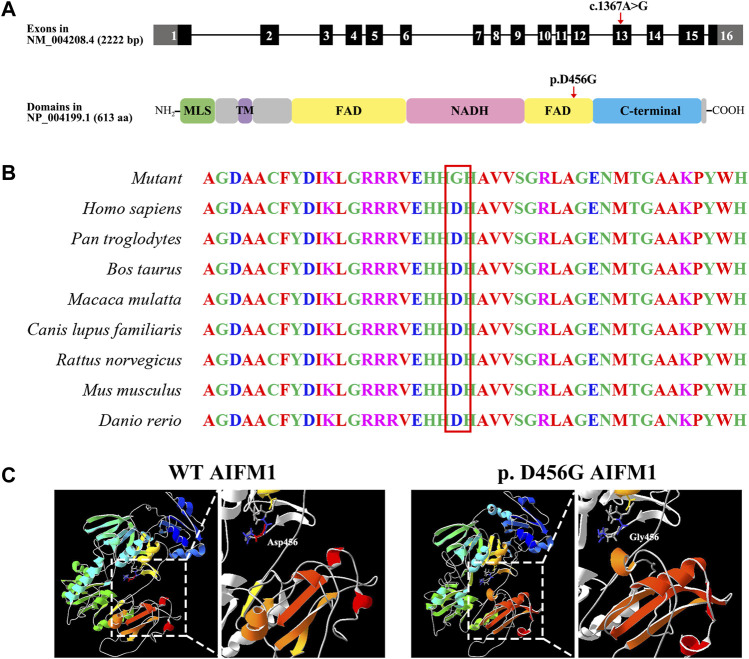
Molecular characteristics of *AIFM1* variant. **(A)** Genomic and protein structures showing the locations of *AIFM1* variant in exon 13 and the FAD domain indicated by the red arrows. The mutation c.1367A > G causes the replacement of individual amino acids (p. D456G) indicated by the red arrow. MLS: Mitochondrial Localization Signal; TM: Transmembrane domain; FAD: Flavin Adenine Dinucleotide; NADH: Nicotinamide Adenine Dinucleotide. **(B)** *AIFM1* protein alignment of eight species of vertebrates showing that the Aspartic (Asp, D) located in 456 highlighted in red box is evolutionarily highly conserved. **(C)** 3D molecular models of the WT and p. D456G *AIFM1* proteins.

As showed in Figure 3A, the variant c.1367A > G was located in exon 13 of the FAD region of *AIFM1*, altering the triplet codon to substitute an aspartic (Asp, D, GAT) with the glycine (Gly, G, GGT) at position 456 (p. D456G). The results of the multiple sequence alignment showed that the *AIFM1* p. D456G occurred at the evolutionarily highly conserved amino acids among eight species of vertebrates (Figure 3B). The 3D structures of WT and p. D456G *AIFM1* were modeled based on the crystal structure of *AIFM1* to reveal that compared with the WT, the structure of p. D456G *AIFM1* protein was evidently changed (Figure 3C) with the polar, acidic amino acid Asp replaced by the non-polar amino acid Gly at position 456 and the secondary structure of the protein changed significantly posterior to the amino acid at position 456.

### Functional analysis of the *AIFM1* variant

**FIGURE 4:**
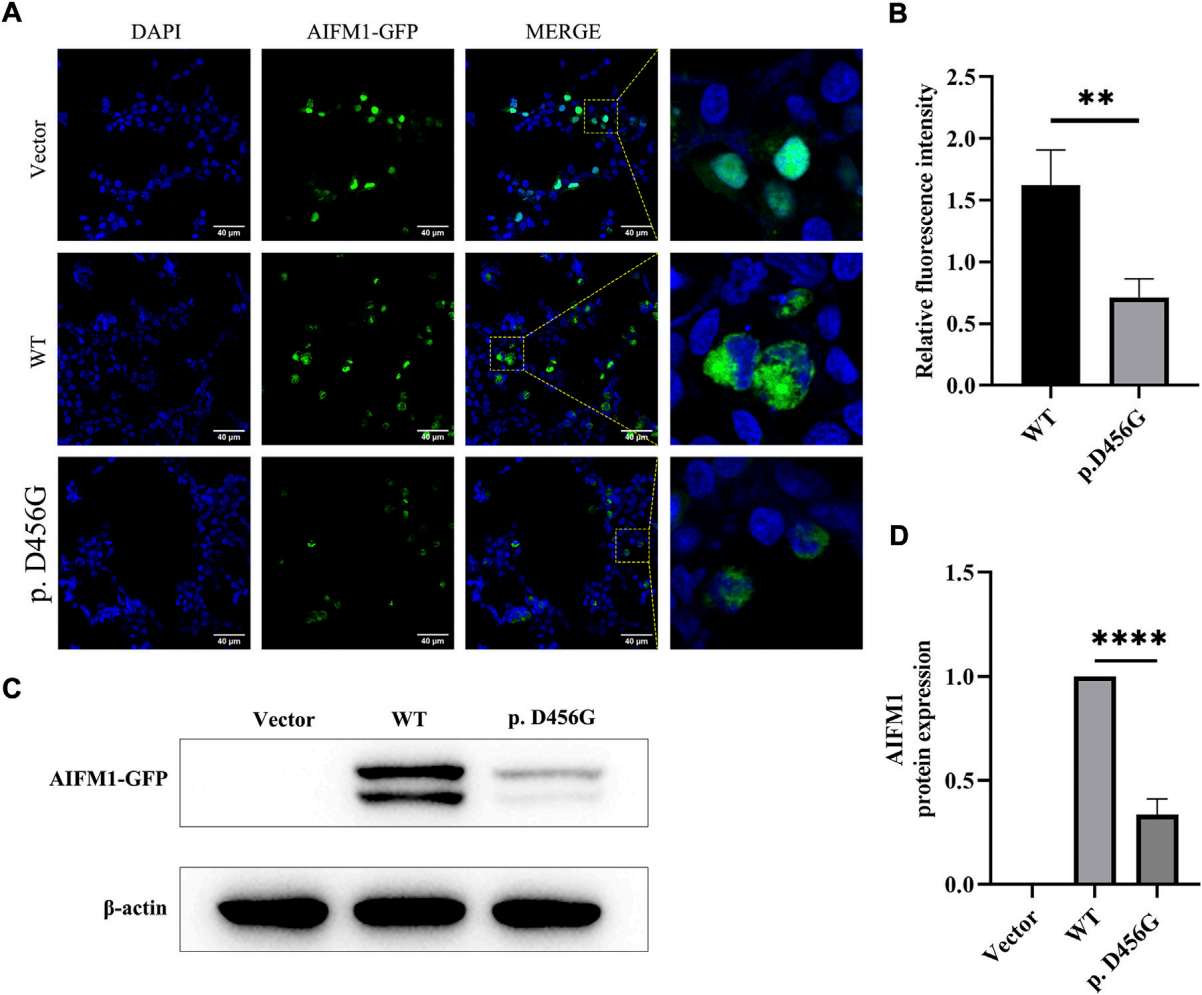
Subcellular localization and protein expression of wild type (WT) and p. D456G *AIFM1* proteins in HEK293 cells. **(A)** Subcellular localization of WT and p. D456G *AIFM1* proteins in HEK293 cells shown in DAPI, GFP-tagged, and merged images. GFP fluorescence is detected after the transient transfection in HEK293 cells. Scale bar = 40 μm. **(B)** Quantitative analysis of fluorescence intensity based on images shown in **(A)**. The data are presented as mean ± standard deviation (SD) of three biological replicates. The significant difference is set at *p* < 0.01 (**) based on the two-tailed unpaired t-test. **(C)** Expressions of WT and p. D456G *AIFM1* in transfected HEK293 cells based on Western blot using anti-GFP and anti-β-actin (i.e., internal control) antibodies. **(D)** Quantitative analysis of proteins shown in **(C).** The data are presented as mean ± standard deviation (SD) of three biological replicates. The significant difference is set at *p* < 0.0001 (****) based on the one-way ANOVA with Dunnett’s multiple comparison tests.

**FIGURE 5:**
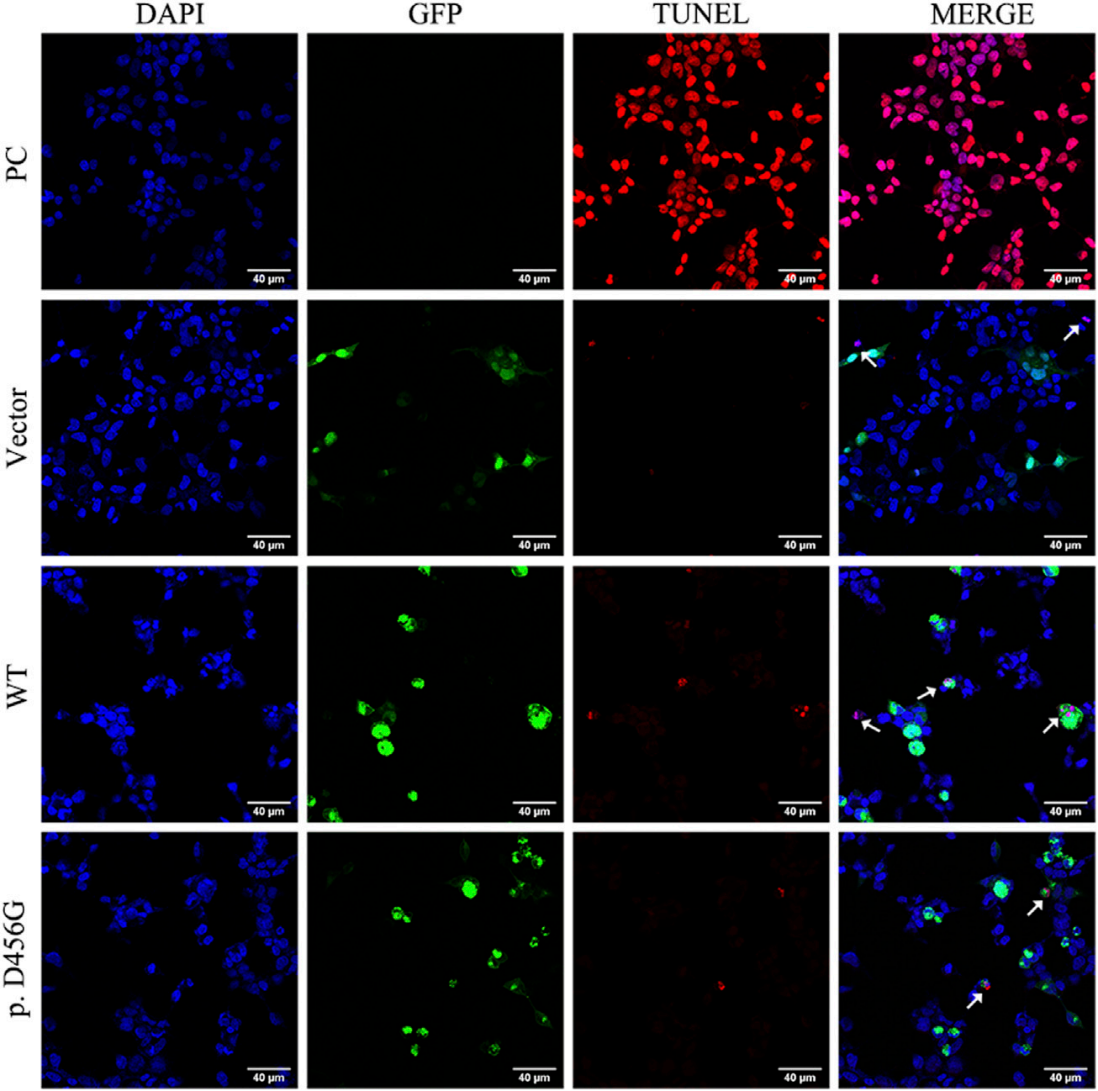
Tunel assay of wild type (WT) and p. D456G *AIFM1* proteins in HEK293 cells shown in Tunel, DAPI, GFP-tagged, and merged images. PC: positive control. The white arrows indicate the apoptotic cells.

After the transfection of WT and mutant *AIFM1* plasmids into HEK293 cells, the subcellular localization of *AIFM1* was detected by GFP fluorescence based on the fusion between the GFPSpark-tag and the C-terminal of *AIFM1* protein. The results showed that green fluorescence was present in the entire cell transfected with vector plasmids and in the cytoplasm of the cells transfected with mutant and WT plasmids, indicating that both the WT and mutant *AIFM1* proteins were localized in the cytoplasm (Figure 4A). Furthermore, immunofluorescence and quantitative results showed that the fluorescence intensity of the p. D456G *AIFM1* was significantly lower than that of the WT *AIFM1* (Figures 4A,B). Western Blot analysis was conducted to further investigate the effects of *AIFM1* variant on the protein expression. The results showed that the *AIFM1* protein expression was not detected in the vector group transfected with vector plasmids, while the WT *AIFM1* protein was detected with the expected molecular weight, suggesting that the staining reaction observed in the immunocytochemical analysis was specifically derived from the GFPSpark-tagged *AIFM1* proteins. The molecular weight of p. D456G *AIFM1* protein was the same as that of the WT *AIFM1* protein but with weaker band than that of the WT *AIFM1* protein (Figure 4C). The quantitative results showed that the expression level of p. D456G *AIFM1* protein was significantly decreased compared with the WT *AIFM1* protein (Figure 4D). In order to investigate the effect of p. D456G on cell survival, we performed the Tunel staining assay (Figure 5). The results showed that compared with the vector group, HEK293 cells transfected with WT *AIFM1* plasmid and c.1367A > G *AIFM1* plasmid showed enhanced apoptosis, showing no significant difference between these two treatments.

## Discussion

As a specific type of hearing loss, AN is characterized by impaired language comprehension. In most cases, *AIFM1* gene is responsible for the cases of late-onset AN (Li et al., 2021), which contributes to the maintenance of normal mitochondrial morphology and physiology as well as caspase-independent apoptosis (Rance and Starr, 2015). In the present study, the possible genetic cause and molecular mechanisms underlying the development of AN were explored in a Chinese family with AN.

The onset ages of patients with late-onset AN are mostly in the range of 5–20 years old with diverse clinical manifestations (Wang et al., 2020). Studies have shown that about 92.5% (360/389) of AN patients are presented with bilateral hearing loss (Bing et al., 2019) with audiogram mainly of low-frequency ascending type and diverse pure tone hearing manifestations which are identified as normal or mild to severe hearing loss (Wang et al., 2003; Wang et al., 2006; Zong et al., 2015; Wang and Starr, 2018), accompanied by significant decline in speech recognition ability (Starr et al., 1996). The results of our study were consistent with the above characteristics reported in the literature, showing that the proband of the family in this study was presented with typical symptoms of late-onset AN, i.e., the onset age of the proband was 15 years old, the audiogram showed low-frequency ascending type (Figure 1C), the PTA showed low-frequency mild hearing loss in both left and right ears with a disproportionate decrease in SDS (Figure 1C), the ABR waveform was illegible (Figure 1B), and the DPOAE was normal (Figure 1E). It was worth noting that although low-frequency ascending type was the most common type of hearing pattern of AN patients, including the patients involved in this study, there are still great differences in hearing patterns among AN patients. For example, a follow-up study found that some AN patients initially showed a low-frequency ascending hearing pattern, but with the progress of the disease, their high-frequency hearing was decreased rapidly to show a descending hearing pattern (Wang et al., 2020). Furthermore, some AN patients present with a descending hearing pattern involving the full frequency were accompanied by severe motor development impairment and mental retardation (Wang et al., 2019). Furthermore, consistent with previous studies (Wang and Starr, 2018), the threshold of ASSR results in our study was also significantly higher than that of PTA (Figure 1D). Moreover, another important clinical manifestation revealed in our study was that the proband complained that his speech recognition ability was worsened in the noisy environment, which was in accordance with the results of a controlled study of AN patients (Lan et al., 2019), suggesting that the speech recognition ability of AN patients with sound speech recognition rate in quiet environment was significantly decreased in the noisy environment. Meanwhile, the proband also showed tinnitus and aggravation under noise environment, which was consistent with the previous study (Xie et al., 2019).

**FIGURE 6:**
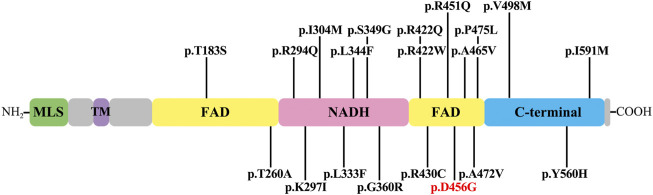
*AIFM1* mutations associated with auditory neuropathy. The novel variant identified in this study is highlighted in red. The structural domains in *AIFM1* shown include mitochondrial localization signal (MLS), transmembrane domain (TM), flavin adenine dinucleotide (FAD), nicotinamide adenine dinucleotide (NADH), and C-terminal domain (C-terminal).

Since the genetic factors account for 42% of the etiology of AN (Moser and Starr, 2016), the genomic DNA of the proband was extracted for NGS analysis. Combined with Sanger sequencing technology, the results showed that the c.1367A > G variant of *AIFM1* carried by the proband came from the mother, and this variant was co-segregated with the phenotype of the family and was not identified in a total of 200 healthy controls. Base on the literature and publicly available databases, the variant c. 1367A > G has not been reported, indicating that this variant of *AIFM1* gene identified in this study was a novel one, expanding the pool of the mutations of *AIFM1* gene. Meanwhile, this variant was predicted as deleterious by REVEL, SIFT, Polyphen-2, and Mutation Taster (Table 1), suggesting the potentially pathogenic property of this variant. To date, a total of 19 pathogenic variants of *AIFM1* related to AN have been reported in literature (Elrharchi et al., 2020; Wang et al., 2020) and databases (Figure 6). The missense mutation (c.1367A > G) revealed in our study provided additional powerful support for molecular diagnosis of AN in clinical settings. These results evidently showed that a novel pathogenic *AIFM1* variant, i.e., a missense mutation (c.1367A > G) in our study was first identified.

The variant of c.1367A > G was located in exon 13 of *AIFM1* gene, causing the amino acid change of p. D456G in the second FAD domain of *AIFM1* protein. Asp is an acidic polar aliphatic amino acid (Agius et al., 2018), which could be used as one of excitation-related neurotransmitter receptors in mammals (Kondoh et al., 2010), while Gly is a kind of non-polar aliphatic amino acid and an inhibitory neurotransmitter in the central nervous system (Roepke et al., 2008). As reported in the previous study (Xu et al., 2017), substitutions of amino acids of different polarities could largely affect the protein structures and functions of target protein. The change from polar Asp to non-polar Gly in this study could largely affect the structure, stability, and activity of the target protein. The 3D protein structure models (Figure 3C) further supported these findings, showing that the secondary and tertiary structures of the protein were largely changed. Studies showed that a total of 19 pathogenic variants previously identified mainly located in the NADH and second FAD domains of *AIFM1* (Elrharchi et al., 2020; Wang et al., 2020), indicating that the NADH and FAD domains play an essential role for the normal function of *AIFM1* protein. The novel variant identified in this study was also located in the second FAD domain (Figure 6), which is required for FAD-dependent NADH oxidoreductase (Zong et al., 2015), suggesting that the novel variant could probably change the redox activity of *AIFM1* to cause abnormal functions of the target protein.

Previous studies showed that changes in key amino acids could affect the conformation of the skeleton and thus disturb the local structure of the protein, even interfere with the inter- and intra-molecular interactions of proteins (Sapra et al., 2008), while *AIFM1* usually interacts with a variety of proteins such as coiled-coil-helix-coiled-coil-helix domain containing 4 (CHCHD4) and Poly (ADP-ribose) polymerase 1 (PARP1). Previous coevolutionary analysis and structural modeling (Hangen et al., 2015; Salscheider et al., 2022) indicated that the N-terminal region of CHCHD4 may form a β-hairpin to bind the C-terminal motif of *AIFM1*. Furthermore, studies showed that *AIFM1* knockdown affected the interaction between CHCHD4 and its substrate and then the biogenesis of electron transport chain, suggesting the essential nature of the interaction between *AIFM1* and CDCDH4 in this process (Salscheider et al., 2022). As shown in Figure 3C, the c.1367A > G variant altered the secondary structure of the C-terminus of *AIFM1*, which may affect its binding to the N-terminus of CDCDH4. PARP1 is a nuclear protein involved in DNA damage, while PAR polymers synthesized by PARP1 bind to *AIFM1* on the mitochondrial membrane with damaged DNA, then *AIFM1* is released to cytoplasm and translocated to the nucleus, resulting in large-scale DNA fragmentation (Wang et al., 2011; Mashimo et al., 2021). Therefore, changes in *AIFM1* protein structure could affect its binding to PAR polymer and thus its ability to induce apoptosis. Furthermore, Asp at position 456 was evolutionarily highly conserved in eight vertebrate species, indicating that amino acid changes may affect protein structure and ultimately protein function. These results indicated that the c.1367A > G variant appeared to be critical for the structures and functions of *AIFM1*.

As an apoptosis-inducing factor, the *AIFM1* is a flavin protein localized in the inner membrane space of mitochondria and is transported from mitochondria to the nucleus to induce cell apoptosis upon the occurrence of the apoptotic injury (Joza et al., 2009). The immunofluorescence analysis showed that the subcellular localization of p. D456G *AIFM1* was consistent with that of the wild-type *AIFM1*, probably due to the MLS (Yuste et al., 2011) and nuclear localization signal (NLS) (Wang et al., 2016) of its N terminus. In a study of *AIFM1* mutation causing Cowchock Syndrome, the mutant *AIFM1* showed more *AIFM1*-positive inclusions detected by immunofluorescence, indicating a higher propensity to translocate to the nucleus (Rinaldi et al., 2012). However, our study showed that there was no significant difference between the mutant group and the wild type group, and the subsequent TUNEL assay showed that p. D456G *AIFM1* and WT *AIFM1* revealed the same TUNEL-positive apoptotic bodies. This difference was probably due to the fact that *AIFM1* could cause a variety of diseases, such as childhood cerebellar ataxia (Heimer et al., 2018), mitochondrial encephalomyopathy (Ghezzi et al., 2010), motor neuropathy (Diodato et al., 2016; Hu et al., 2017; Sancho et al., 2017), Cowchock syndrome (Rinaldi et al., 2012) and auditory neuropathy (Zong et al., 2015; Wang et al., 2020), with different severity and potential pathogenesis.

Furthermore, the immunofluorescence analysis also showed that the fluorescence intensity of p. D456G *AIFM1* was weaker than that of WT *AIFM1*, indicating that the variant of p. D456G changed the expression level of *AIFM1*. To further investigate the effect of p. D456G mutation on *AIFM1* protein expression, western blot analysis was performed. The results were consistent with those of the immunofluorescence analysis, showing that the protein expression of p. D456G *AIFM1* was significantly lower than that of wild-type *AIFM1*. As predicted by our 3D protein model, the p. D456G mutation affected the secondary/tertiary structure of the protein, resulting in degradation or low expression of the protein, which was further supported by the results of western blot. Previously, many scholars have studied the effect of *AIFM1* mutation on protein expression with varied results. For example, patients with p. G338E (Diodato et al., 2016) and p. F210S (Sancho et al., 2017) variants all showed motor nerve lesions, and the expression level of *AIFM1* was decreased. These results were consistent with the findings revealed in our study. Interestingly, in a study on axonal polyneuropathy caused by *AIFM1* p. F210L variant (Hu et al., 2017), no significant change was detected in the *AIFM1* protein expression, independent of the changes of different amino acids at the same site. Furthermore, in a study of mitochondrial encephalomyopathy caused by R201 del variant in *AIFM1*, the protein expression was not significantly altered (Ghezzi et al., 2010).

Under normal circumstances, *AIFM1* is first transcribed and translated into a 67-kD precursor molecule (Liu et al., 2018), which is then transported to mitochondria through the N-terminal domain MLS sequence. The mitochondrial processing peptidase (MPP) cleaves *AIFM1* in mitochondria into 62-kD mature *AIFM1*, which acts as the FAD-dependent NADH oxidoreductase to contribute to the stabilization and maturation of mitochondrial oxidation of respiratory chain complex I as well as the removal of peroxide from the mitochondria (Susin et al., 1999). When cells are damaged by apoptosis, mature *AIFM1* is cleaved into soluble apoptotic precursor protein, i.e., the truncated AIF (tAIF) of about 57 kD (Susin et al., 1999; Joza et al., 2001). The tAIF is released from mitochondria into cytosol and nucleus to induce two typical caspase-independent apoptosis phenomena: chromatin condensation and fragmentation of large DNA fragments of approximately 50 kD (Yuste et al., 2011).

A study of *AIFM1* p. P488L variants in auditory neuropathy and peripheral neuropathy showed that *AIFM1* p. P488L variants caused a mild increase in the rate of caspase-independent apoptosis in cells (Wang et al., 2019). Because the immunofluorescence and WB assays showed that the p. D456G variant reduced *AIFM1* protein expression, the Tunel assay was performed to evaluate its adverse effect on cell survival. The results showed that the ability of *AIFM1* to induce apoptosis was not significantly affected by the p. D456G mutation, showing the same effect on cell survival as that of WT AIFM, indicating that the p. D456G variant did not affect cell survival. This may also be the reason for the generation of mild symptoms of AN patients. Previous studies showed that the *AIFM1* R201del variant caused severe mitochondrial encephalomyopathy and significantly increased parthanatos-linked cell death (Ghezzi et al., 2010). In the study of Cowchock Syndrome caused by *AIFM1* p. E493V variant, Tunel assay of muscle biopsy showed a large number of apoptotic cells in the samples of the mutated individuals, while little or no staining was detected in the muscles of healthy controls (Rinaldi et al., 2012). However, in patients with distal motor neuropathy carrying the p. F210S mutation, the mutation did not enhance the apoptosis (Sancho et al., 2017), which was consistent with our study. Similarly, as mentioned above, the p. F210S mutation also reduced the protein expression level with mitochondrial fragmentation also observed in fibroblasts of this patient, suggesting that the development of distal motor neuropathy in this patient may be related to the defective mitochondrial respiration (Sancho et al., 2017). Therefore, we hypothesized that this mutation might affect the role of *AIFM1* in oxidative phosphorylation, redox control, and respiratory chain activity. Study showed that knockdown of *AIFM1* could attenuate mitochondrial respiration and ATP production, ultimately affecting cell functions (Zong et al., 2020). Furthermore, some studies showed that in the auditory pathway, including inner hair cells, glial cells in neural pathways, and spiral ganglion cell (SGC), the normal energy metabolism was the key to maintain its physiological activity (Yang et al., 2015). Therefore, as suggested in the previous studies (Zong et al., 2015), the variant revealed in our study may cause AN by affecting the function of *AIFM1* protein in mitochondria, ultimately affecting the mitochondrial respiration and ATP synthesis in inner ear. Moreover, as mentioned above, the 3D prediction results showed that the p. D456G mutation affected both the secondary and tertiary structures of the C-terminal, while the binding of the N-terminal of CHCHD4 and the C-terminal of *AIFM1* played an important role in the biogenesis of mitochondrial respiratory chain complex Ⅰ. The decreased expression level of *AIFM1* could affect this interaction, and then affect the function of the mitochondrial respiratory chain. These functional experiments further demonstrated that the novel mutation identified in this study could cause damage to *AIFM1* protein, which could be the underlying genetic etiology of this family with AN, and further experiments of mitochondrial respiratory chain activity and oxidative phosphorylation are necessary to explore the pathological mechanism of this mutation.

In summary, we identified a novel c.1367A > G *AIFM1* variant in a Chinese family with AN by targeted capture sequencing, expanding the AN-related mutation spectrum of *AIFM1*. Bioinformatics prediction and functional analysis showed that *AIFM1* c.1367A > G was a pathogenic mutation and may be the genetic cause of AN in this Chinese family, providing additional molecular and clinical evidence to support the establishment of a strong genotype-phenotype correlation for AN.

## Data Availability

The data presented in the study are deposited in the the NCBI Sequence Read Archive (SRA) repository, accession number PRJNA891130.
